# Environmental Chemicals in Urine and Blood: Improving Methods for Creatinine and Lipid Adjustment

**DOI:** 10.1289/ehp.1509693

**Published:** 2015-07-24

**Authors:** Katie M. O’Brien, Kristen Upson, Nancy R. Cook, Clarice R. Weinberg

**Affiliations:** 1 Biostatistics and Computational Biology Branch, and; 2 Epidemiology Branch, National Institute of Environmental Health Sciences, National Institutes of Health, Department of Health and Human Services, Research Triangle Park, North Carolina, USA; 3 Division of Preventive Medicine, Brigham and Women’s Hospital, Harvard Medical School, Boston, Massachusetts, USA

## Abstract

**Background:**

Investigators measuring exposure biomarkers in urine typically adjust for creatinine to account for dilution-dependent sample variation in urine concentrations. Similarly, it is standard to adjust for serum lipids when measuring lipophilic chemicals in serum. However, there is controversy regarding the best approach, and existing methods may not effectively correct for measurement error.

**Objectives:**

We compared adjustment methods, including novel approaches, using simulated case–control data.

**Methods:**

Using a directed acyclic graph framework, we defined six causal scenarios for epidemiologic studies of environmental chemicals measured in urine or serum. The scenarios include variables known to influence creatinine (e.g., age and hydration) or serum lipid levels (e.g., body mass index and recent fat intake). Over a range of true effect sizes, we analyzed each scenario using seven adjustment approaches and estimated the corresponding bias and confidence interval coverage across 1,000 simulated studies.

**Results:**

For urinary biomarker measurements, our novel method, which incorporates both covariate-adjusted standardization and the inclusion of creatinine as a covariate in the regression model, had low bias and possessed 95% confidence interval coverage of nearly 95% for most simulated scenarios. For serum biomarker measurements, a similar approach involving standardization plus serum lipid level adjustment generally performed well.

**Conclusions:**

To control measurement error bias caused by variations in serum lipids or by urinary diluteness, we recommend improved methods for standardizing exposure concentrations across individuals.

**Citation:**

O’Brien KM, Upson K, Cook NR, Weinberg CR. 2016. Environmental chemicals in urine and blood: improving methods for creatinine and lipid adjustment. Environ Health Perspect 124:220–227; http://dx.doi.org/10.1289/ehp.1509693

## Introduction

In epidemiologic studies of environmental contaminants measured in urine, investigators adjust for creatinine or specific gravity to correct for variations in urine diluteness at the time of measurement (Barr et al. 2005; Thorne 2008). Similarly, contaminant concentrations for lipophilic chemicals measured in blood are adjusted for serum lipid level (SLL) (Phillips et al. 1989; Schisterman et al. 2005).

Most investigators agree that adjustment is beneficial, but controversy has arisen regarding the best approach (Schisterman et al. 2005). Traditionally, investigators standardize measured urinary biomarker concentrations by dividing by the concentration of urinary creatinine. This division converts the scale to weight of chemical per weight creatinine, reflecting the assumption that creatinine excretion is approximately constant across individuals and time. In principle, because individuals with low urinary creatinine concentrations are well hydrated, they would have commensurately dilute urinary concentrations of environmental contaminants. Thus, standardization would equalize concentrations across individuals and across time within individuals. The same concept applies to adjustment for SLL because individuals with elevated lipid concentrations tend to carry proportionally higher concentrations of lipid-soluble contaminants (Longnecker et al. 1996; Phillips et al. 1989).

Schisterman et al. (2005) challenged this classical standardization approach by demonstrating its poor performance in simulated scenarios involving lipophilic chemicals measured in serum. Considering simulations based on a number of directed acyclic graphs (DAGs), the authors found that simply including serum lipid as a covariate in the regression model generated more accurate and precise effect estimates than traditional standardization. They also demonstrated good performance of a two-stage model in which SLL was regressed on the contaminant (stage I) with the resulting residual term then entered as a covariate when modeling the effect of the contaminant on the outcome (stage II) (Hunter et al. 1997). The paper by Schisterman et al. (2005) has been widely cited, reflecting its substantial influence on analytic practice. However, we believe that some important causal scenarios remain to be explored.

To set the stage for our alternative scenarios, consider the purpose for which urinary and blood measurements are made. In many applications, urine and blood are used as accessible proxies for inaccessible target tissues. For example, when examining the effects of bisphenol A (BPA) on breast cancer, breast and reproductive organs are probably the most disease-relevant tissues. Instead, however, we measure urinary BPA concentration, not because the urine itself is a source of exposure, but as a surrogate. Because the target and proxy contaminant concentrations can differ, the use of proxy measurements results in measurement error, which causes bias. Furthermore, because the target and the proxy have different relationships with the outcome and with other factors, the choice of confounding variables may depend on how the causal network is defined.

A further complication to identifying true exposure levels is that risk factors for the disease under study might also affect creatinine or SLL. For example, creatinine levels can vary by sex, race, age, fat-free mass, and body mass index (BMI) (Barr et al. 2005). Sex, age, and BMI are also associated with SLL (Costanza et al. 2005). Barr et al. (2005) consequently recommended adjusting for creatinine as a covariate in the regression model. However, according to DAG theory (Greenland et al. 1999), creatinine (or SLL) may act as a “collider,” that is, a common descendant of two other variables on a causal pathway. If so, epidemiologists have demonstrated that adjusting for creatinine (or SLL) could induce noncausal associations and lead to further confounding (Cole et al. 2010). Additionally, adjusting for creatinine or serum lipids as covariates may not adequately control for the measurement error that results from between-subject variations in urinary dilution or SLL. That issue is considered in the present study with the aid of DAGs.

We also consider how to control for measurement error when using proxy biomarker measures. We demonstrate the limitations of existing approaches and propose novel methods to control confounding and measurement error more effectively. We construct DAGs corresponding to several scenarios with distinct causal frameworks for toxicants measured in urine (Part I) versus those measured in serum (Part II). For each setting, we present results from simulation studies conducted to compare methods. In Part III, we apply these approaches to real data in a study of urinary phthalate concentrations and early pregnancy loss.

## Part I: Environmental Chemicals Measured in Urine

**Figure 1:**
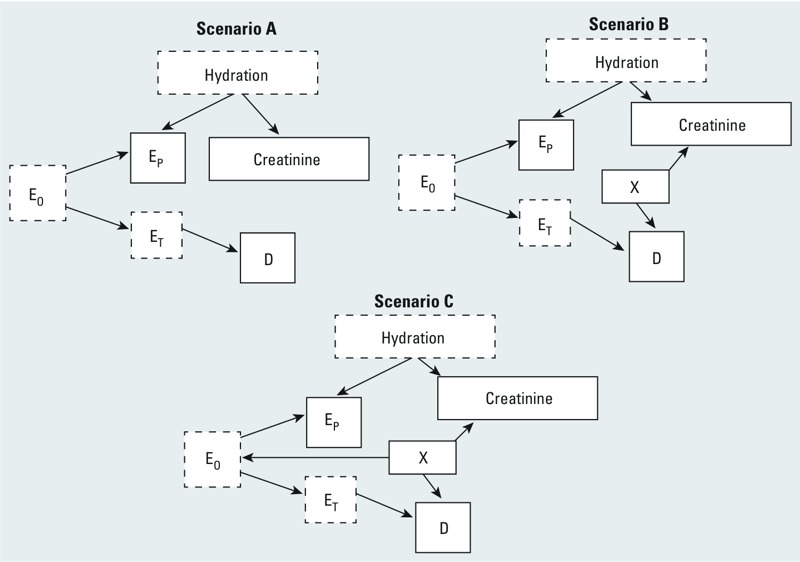
Directed acyclic graphs illustrating three possible relationships (scenarios A–C) among overall exposure concentrations (E_O_), target-tissue exposure concentrations (E_T_), urinary (proxy) exposure concentrations (E_p_), hydration, creatinine concentration, covariate X, and disease (D). Variables with solid outlines are observed, those with dashed outlines are unobserved.

*Methods.* We first consider scenarios where urine serves as a proxy for disease-relevant tissues (Figure 1, DAGs A–C). For instance, suppose we want to measure the association between breast cancer risk and BPA concentrations in breast tissue (i.e., target tissue, presumably causal), but we can measure only urinary BPA concentrations (presumably not causal). For the sake of simplicity, we assume that overall exposure and the consequent target tissue biomarker concentrations are stable across time and ignore the error caused by obtaining a “snapshot” measurement rather than measuring cumulative exposure. Our only focus is on the part of measurement error that adjustment for creatinine in the urine sample can potentially mitigate, that is, the discrepancy between urinary and target tissue concentrations at the time that the proxy sample was collected.

In scenario A (Figure 1), target-tissue concentrations (E_T_), which depend on overall environmental exposure (E_O_), affect disease risk. Proxy concentrations (E_P_), measured in urine, depend on both E_O_ and hydration levels at the time of sample collection. Hydration commensurately affects creatinine levels. Scenario B (Figure 1) additionally allows some covariate X (e.g., age) to affect both creatinine and disease risk. Conditioning on urinary creatinine by adjusting for it in the model will induce an association between E_P_ and disease (Cole et al. 2010; Greenland et al. 1999) unless X is also included in the model. Scenario C (Figure 1) is similar to scenario B except that X can also affect E_O_. Conditioning on creatinine will again open a “back-door path” between E_P_ and disease unless one also adjusts for X.

For the simulation study, we generated data for each of the relevant covariates by randomly drawing values from specified distributions. Our primary purpose for doing so was to compare the effect estimates produced by an analysis of the simulated data to the true effect estimates, which we defined when designing the simulation. We selected five possible values of the true odds ratio (OR, per unit change) for the effect of E_T_ on disease: 2.00, 1.30, 1.00, 0.77, and 0.50. These values correspond to true natural log (ln) OR values (denoted β_TRUE_) of 0.69, 0.26, 0.00, –0.26, and –0.69, respectively. Each simulation was repeated 1,000 times. The sample included 500 participants when the OR was 2.0 or 0.5 and 1,000 otherwise.

The variable distributions selected for the simulation study are discussed in detail in Supplemental Material, “Part I: Description of simulation study parameters for urine biomarker scenarios (DAGs A–C).” Briefly, we generated values for urinary biomarker concentrations with a log-normal distribution that approximates the distribution seen for BPA in female participants from the 2007–2010 National Health and Nutrition Examination Surveys [NHANES; Centers for Disease Contol and Prevention (CDC) 2009, updated 2013]. We simulated values for creatinine, hydration, and X based on the assumptions specified in the DAG. In sensitivity analyses, we simulated assay-specific measurement errors by including a random error term in the equation used to generate E_P_ or creatinine. All analyses were performed in SAS (version 9.3; SAS Institute Inc., Cary, NC).

The presence or absence of disease was assigned by random draws from a binomial distribution where the ln odds of having disease (D) was linearly dependent on the target-tissue concentrations, E_T_: logit[Pr(D)] = α + β_TRUE_ × E_T_ + δ × W. Here, W is a vector containing any relevant confounders. We selected intercept terms to impose case–control sampling with approximately 50% cases.

To enable scale-invariant comparisons between results based on E_T_ versus E_P_, we rescaled the biomarker measures using standardized *z*-scores. Then, for each of seven statistical approaches described below, we estimated the association between one standard deviation (SD) increase in E_P_ (with or without prior creatinine standardization, depending on the approach) and the change in ln odds of disease risk. The resulting estimated coefficient for the E_P_ *z*-score (E_Pz_), should be very close to the corresponding β for the E_T_ *z*-score. The derivations of the coefficients for the E_T_ *z*-scores (E_Tz_) corresponding to each DAG are defined in Supplemental Material, Table S1, “Variable relationships, urinary biomarker scenarios (A–C),” and are listed in the results tables. To the extent that the estimated coefficient for E_Pz_ systematically differs across simulations from the true coefficient for E_Tz_, there is bias.

Method 1. We fit a model that does not adjust for creatinine: logit[Pr(D)] = α + β × E_Pz_ + δ × W. This naïve approach illuminates the consequences of ignoring dilution effects. When analyzing scenarios B and C, we adjusted for factor X as a confounder. This adjustment was made in all seven approaches, which are described in detail in Table 1.

**Table 1:** Statistical models for each analytic method, as applied to biomarkers measured in urine.

Method	logit[Pr(D)] =
1. Unadjusted	α + β × E_Pz_ + δ × W
2. Standardized	α + β × ratio_z_ + δ × W
3. Covariate-adjusted standardization	α + β × Cratio_z_ + δ × W
4. Covariate adjustment	α + β × E_Pz_ + λ × creatinine + δ × W
5. 2-stage model	α + β × E_Pz_ + θ × R + δ × W; creatinine = α + β × E_Pz_ + R
6. Standardization plus covariate adjustment	α + β × ratio_z_ + λ × creatinine + δ × W
7. Covariate-adjusted standardization plus covariate adjustment	α + β × Cratio_z_ + λ × creatinine + δ × W
Abbreviations: $\hat{\mathrm{Cr}}$, predicted creatinine; Cratio, $E_{P}/(\mathrm{Cr}/\hat{\mathrm{Cr}})$; E_P_,proxy exposure level; E_Pz_, proxy exposure *z*-score; ratio_z_, *z*-score for E_P_:creatinine ratio. Creatinine predicted based on X_1_ in all scenarios. W not included in Scenario A. In scenarios B and C, W = X_1_.	

Method 2. We compute the ratio of E_P_ to creatinine and then estimate the effect per SD: logit[Pr(D)] = α + β × ratio_z_ + δ × W. This is the commonly used creatinine standardization method, which reflects the assumption that creatinine levels are inversely proportional to urinary diluteness.

Method 3. The third approach, covariate-adjusted standardization, allows for systematic differences in long-term average creatinine levels across subpopulations. We first fit a model for ln(creatinine) as a function of the covariates thought to directly and chronically affect it (e.g., factor X). We then standardize by calculating Cratio = E_P_/(Cr/C^^^^^r), where Cr and C^^^^^r denote the observed and fitted creatinine, respectively. Finally, we standardize Cratio and fit: logit[Pr(D)]= α + β × Cratio_z_ + δ × W. This method should specifically control the covariate-independent, short-term multiplicative effect of hydration on urinary diluteness.

Method 4. The fourth approach includes creatinine in the model: logit[Pr(D)] = α + β × E_Pz_ + λ × creatinine + δ × W. As discussed previously, the inclusion of creatinine serves to block confounding causal pathways involving both creatinine and disease (Barr et al. 2005; Schisterman et al. 2005).

Method 5. The fifth approach uses the two-stage model suggested by Schisterman et al. (2005), first modeling creatinine as if affected by E_Pz_: creatinine = α + β × E_Pz_ + R, and then including the residual (R) in the model: logit[Pr(D)] = α + β × E_Pz_ + θ × R + δ × W.

Methods 6 and 7. The final two approaches are motivated by scenario C. We use the standardized biomarker measure (ratio_z_, as in Method 2) or the covariate-adjusted standardized biomarker measure (Cratio_z_, as in Method 3) in regression models that also include creatinine as a covariate: logit[Pr(D)] = α + β × ratio_z_ + λ × creatinine + δ × W (Method 6) or logit[Pr(D)] = α + β × Cratio_z_ + λ × creatinine + δ × W (Method 7). The goals of these methods are to control for variation due to hydration and to reduce confounding by blocking back-door paths between creatinine and risk factors related to both creatinine and disease. As with Method 3, Method 7 should allow separate control for the independent, multiplicative effect of hydration on diluteness.

For each of the seven methods and 1,000 data simulations, we obtained a point estimate and a variance estimate for the coefficient of the urinary biomarker measure of interest (E_Pz_, ratio_z_ or Cratio_z_). To measure bias, we subtracted the true beta coefficient for E_Tz_ [i.e., the ln(OR), which corresponds to the standardized concentration in the target tissue as specified for that simulation] from the mean of the 1,000 point estimates. We also calculated the square root of the mean of the 1,000 estimated variances across all simulations as well as the empirical SD, which is the SD of the 1,000 point estimates. Concordance between these values indicates good model-based variance estimation at the simulated sample size. We also calculated the empirical confidence interval (CI) coverage, which is the proportion of simulations in which the 95% confidence interval included the true beta coefficient of E_Tz_. The standard error of the bias was calculated by dividing the empirical SD by the square root of the number of simulations (*n* = 1,000).

*Results.* Table 2 shows the results of the simulations. The effect estimates are marked with an asterisk (*) if CI coverage was statistically consistent with 95% (0.95 ± 0.0135).

**Table 2:** Results from simulation studies comparing seven methods for creatinine adjustment when assessing the relationship between a urinary biomarker and disease risk under different causal scenarios (Figure 1) and true effect sizes (true ORs = 2.0, 1.3, 1.0, 0.77, or 0.5).

Analysis method	Scenario A		Scenario B		Scenario C	
	Bias (SE)^*a*^	CI coverage	Bias (SE)^*a*^	CI coverage	Bias (SE)^*a*^	CI coverage
True OR = 2.0, true β for E_Tz _= 0.650 (A and B) or 0.690 (C)
1. Unadjusted	–0.02 (0.003)	0.92	–0.02 (0.003)	0.93	–0.03 (0.004)	0.93
2. Standardized^*b*^	0.01 (0.003)	0.93	0.08 (0.004)	0.90	0.17 (0.005)	0.82
3. Covariate-adjusted standardization (CAS)^*b*^^,^^*c*^	0.01 (0.003)	0.93	0.01 (0.003)	0.94*	0.00 (0.004)	0.94
4. Covariate adjustment (CA)^*b*^	0.03 (0.003)	0.92	0.03 (0.004)	0.94*	0.02 (0.004)	0.93
5. 2-stage model^*b*^	–0.01 (0.003)	0.91	–0.01 (0.003)	0.93	0.07 (0.004)	0.92
6. Standardization plus CA^*b*^	0.01 (0.003)	0.93	0.08 (0.004)	0.90	0.17 (0.005)	0.81
7. CAS plus CA^*b*^^,^^*c*^	0.01 (0.003)	0.93	0.01 (0.003)	0.94*	0.01 (0.004)	0.94*
True OR = 1.3, true β for E_Tz_ = 0.245 (A and B) or 0.260 (C)
1. Unadjusted	–0.01 (0.002)	0.95*	–0.01 (0.002)	0.94*	–0.01 (0.002)	0.94*
2. Standardized^*b*^	0.00 (0.002)	0.95*	0.03 (0.002)	0.93	0.06 (0.003)	0.90
3. CAS^*b*^^,^^*c*^	0.00 (0.002)	0.95*	0.00 (0.002)	0.95*	0.00 (0.002)	0.95*
4. CA^*b*^	0.01 (0.002)	0.95*	0.01 (0.002)	0.94*	0.01 (0.002)	0.95*
5. 2-stage model^*b*^	–0.01 (0.002)	0.95*	–0.01 (0.002)	0.94*	0.03 (0.003)	0.95*
6. Standardization plus CA^*b*^	0.00 (0.002)	0.95*	0.03 (0.002)	0.93	0.06 (0.003)	0.90
7. CAS plus CA^*b*^^,^^*c*^	0.00 (0.002)	0.95*	0.00 (0.002)	0.94*	0.00 (0.002)	0.95*
True OR = 1.0, true β for E_Tz_ = 0.0
1. Unadjusted	0.00 (0.002)	0.96*	0.00 (0.002)	0.96*	0.00 (0.002)	0.96*
2. Standardized^*b*^	0.00 (0.002)	0.95*	0.00 (0.002)	0.96*	0.00 (0.003)	0.96*
3. CAS^*b*^^,^^*c*^	0.00 (0.002)	0.95*	0.00 (0.002)	0.95*	0.00 (0.002)	0.95*
4. CA^*b*^	0.00 (0.002)	0.95*	0.00 (0.002)	0.95*	0.00 (0.002)	0.95*
5. 2-stage model^*b*^	0.00 (0.002)	0.95*	0.00 (0.002)	0.95*	0.00 (0.002)	0.96*
6. Standardization plus CA^*b*^	0.00 (0.002)	0.95*	0.00 (0.002)	0.96*	0.00 (0.003)	0.96*
7. CAS plus CA^*b*^^,^^*c*^	0.00 (0.002)	0.95*	0.00 (0.002)	0.95*	0.00 (0.002)	0.95*
True OR = 0.77, true β for E_Tz_ = –0.245 (A and B) or –0.260 (C)
1. Unadjusted	0.01 (0.002)	0.95*	0.01 (0.002)	0.95*	0.01 (0.002)	0.96*
2. Standardized^*b*^	0.00 (0.002)	0.96*	–0.02 (0.002)	0.94*	–0.04 (0.003)	0.94
3. CAS^*b*^^,^^*c*^	0.00 (0.002)	0.96*	0.00 (0.002)	0.95*	0.00 (0.002)	0.96*
4. CA^*b*^	–0.01 (0.002)	0.95*	–0.01 (0.002)	0.95*	–0.01 (0.002)	0.96*
5. 2-stage model^*b*^	0.01 (0.002)	0.95*	0.01 (0.002)	0.95*	–0.02 (0.002)	0.95*
6. Standardization plus CA^*b*^	0.00 (0.002)	0.96*	–0.02 (0.002)	0.94*	–0.04 (0.003)	0.94*
7. CAS plus CA^*b*^^,^^*c*^	0.00 (0.002)	0.96*	0.00 (0.002)	0.95*	0.00 (0.002)	0.96*
True OR = 0.5, true β for E_Tz _= –0.650 (A and B) or –0.690 (C)
1. Unadjusted	0.02 (0.003)	0.94*	0.02 (0.003)	0.92	0.00 (0.004)	0.94*
2. Standardized^*b*^	–0.01 (0.003)	0.95*	–0.06 (0.004)	0.92	–0.02 (0.004)	0.87
3. CAS^*b*^^,^^*c*^	–0.01 (0.003)	0.95*	0.00 (0.003)	0.94*	–0.01 (0.004)	0.95*
4. CA^*b*^	–0.03 (0.003)	0.94*	–0.02 (0.004)	0.95*	–0.02 (0.004)	0.95*
5. 2-stage model^*b*^	0.01 (0.003)	0.94*	0.02 (0.003)	0.92	0.01 (0.004)	0.91
6. Standardization plus CA^*b*^	–0.01 (0.003)	0.95*	–0.06 (0.004)	0.92	–0.01 (0.004)	0.87
7. CAS plus CA^*b*^^,^^*c*^	–0.01 (0.003)	0.95*	0.00 (0.003)	0.94*	–0.02 (0.004)	0.95*
Abbreviations: E_Tz_, target-tissue exposure *z*-score; E_Pz_, proxy exposure *z*-score; SE, standard error; CI, confidence interval. Each simulation was repeated 1,000 times. Samples included 500 observations when the true OR = 2.0 or 0.5 and 1,000 observations otherwise. ^***a***^Bias is equal to the mean observed beta coefficient for β_Pz_, which is either the urine exposure *z*-score (Methods 1, 4, 5) or the *z*-score for the urine exposure:creatinine ratio (Methods 2, 3, 6), minus the true beta coefficient for E_Tz_. The standard error of the bias estimate is the square root of the average variance of β_Pz_ divided by the square root of the number of simulations. ^***b***^B and C are adjusted for X. ^***c***^Creatinine is predicted using X. *CI coverage is consistent with 0.95 (0.95 ± 0.0135). Note that CI coverage values are rounded, and only those values that are consistent with 95% have been marked with an asterisk.						

For scenario A, Methods 1 (unadjusted) and 4 (covariate-adjusted) were biased relative to the other methods when E_T_ had an effect (i.e., when the true OR ≠ 1.0). The other methods performed very well, with little to no detectable bias (± 0.01), and CI coverage consistent with 95% except when the OR = 2.0, in which case CI coverage was consistently < 95%. The values of the model-based SDs were, on average, close to those of the empirical SDs across all scenarios and statistical approaches (data not shown).

For scenario B, the covariate-adjusted standardization methods (3 and 7) performed consistently well. The other methods were more biased, especially the traditional standardization methods (2 and 6). Here, when the true OR was 2.0, the bias was 0.08, which corresponds to a change of 12% (0.08/E_Tz_ = 0.08/0.65). CI coverages were consistent with 95% for Methods 3, 4, and 7 under all scenarios.

For scenario C, when the effect was large and positive (true OR = 2.0), the traditional standardization approaches were highly biased (0.17 = 26% change for Methods 2 and 6). Methods 3 and 7 had consistently low bias and CI coverages near 95%.

When there was no true effect (true OR = 1.0), all seven methods showed CI coverages consistent with 95% and minimal bias for all three scenarios. Thus, all seven approaches provide valid hypothesis tests. When there was a true effect, however, only the covariate-adjusted standardized approaches (3 and 7) performed well under all scenarios, with CI coverage consistent with 95%.

Results for scenarios with classical assay measurement error introduced for both E_P_ and creatinine are shown in Supplemental Material, Table S2, “Results from simulations with measurement error: urinary biomarker scenarios (A–C).” As expected, the estimates were generally more biased than when E_P_ and creatinine were measured without error. Although the seven methods varied in their relative performance, the covariate-adjusted standardization plus creatinine adjustment method (7) again performed well, showing minimal bias and good coverage for all but one of the six tested methods.

In general, we found that both covariate-adjusted standardization approaches (Methods 3 and 7) performed well in all simulation scenarios and effect size specifications, with minimal bias and close to nominal CI coverage rates. This good performance persisted even when there was a complicated confounding structure and simulated laboratory measurement error. Because real-life scenarios will likely involve more complicated causal structures than those modeled here, Method 7 (covariate-adjusted standardization plus creatinine adjustment) may have better general utility.

## Part II: Environmental Chemicals Measured in Serum

**Figure 2:**
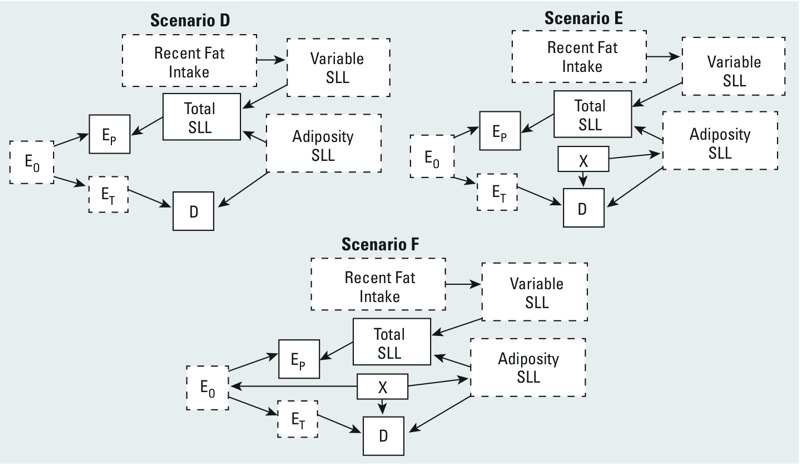
Directed acyclic graphs illustrating three possible relationships (scenarios D–F) among overall exposure concentrations (E_O_), target-tissue exposure concentrations (E_T_), serum (proxy) exposure concentrations (E_p_), recent fat intake, adiposity serum lipid levels (SLL), variable SLL, total SLL, covariate X, and disease (D). Variables with solid outlines are observed, those with dashed outlines are unobserved.

*Methods.* We next consider scenarios where lipophilic chemicals are measured in serum (Figure 2, DAGs D–F). To parallel the previous example, suppose we want to measure the association between a biomarker in a target tissue [e.g., polychlorinated biphenyl (PCB) in breast tissue] and an outcome (e.g., breast cancer), but only measure PCB concentrations in serum. For the sake of simplicity, we again assume that exposure concentrations are stable across time and focus on the measurement error that we can potentially adjust for by accounting for SLL at the time of sample collection. Further details of the serum biomarker simulation study are discussed in Supplemental Material, “Part II: Description of simulation study parameters for serum biomarker scenarios (DAGs D–F).”

In these scenarios, total SLL is the sum of two components: adiposity-related SLL and variable SLL. Adiposity SLL is positively associated with obesity and is temporally stable. In contrast, variable SLL changes after recent fat intake (Longnecker et al. 1996; Phillips et al. 1989).

In all three scenarios, total SLL affects the serum concentration of lipid-soluble contaminants (E_P_), and adiposity SLL is associated with disease risk, as is the target-tissue biomarker concentration (E_T_). In scenarios E and F, we also assume that factor X (e.g., BMI) affects both adiposity SLL and disease risk. In scenario F, X also affects overall exposure [E_O_; see Supplemental Material, Table S3, “Variable relationships, serum biomarker scenarios (D–F)”].

We used the same E_O_ distribution as that used in the previous example and simulated 1,000 case–control studies with five possible true effect parameters (2.00, 1.30, 1.00, 0.77, and 0.50), setting the sample size to 500 when the OR was 2.0 or 0.5 and to 1,000 otherwise. For sensitivity analyses of assay-specific measurement error, we added a random error term to the equation used to generate E_P_ or total SLL [see Supplemental Material, “Part II: Description of simulation study parameters for serum biomarker scenarios (DAGs D–F)”]. Further details of the seven statistical approaches applied to serum biomarkers are shown in Table 3.

**Table 3:** Statistical models for each analytic method, as applied to biomarkers measured in serum.

Method	logit[Pr(BC)] =
1. Unadjusted	α + β × E_Pz_ + δ × W
2. Standardized	α + β × ratio_z_ + δ × W
3. Covariate-adjusted standardization	α + β × Cratio_z_ + δ × W
4. Covariate adjustment	α + β × E_Pz_ + λ × SLL + δ × W
5. 2-stage model	α + β × E_Pz_ + θ × R + δ × W; SLL = α + β × E_Pz_ + R
6. Standardization plus covariate adjustment	α + β × ratio_z_ + λ × SLL + δ × W
7. Covariate-adjusted standardization plus covariate adjustment	α + β × Cratio_z_ + λ × SLL + δ × W
Abbreviations: Cratio, $E_{P}/(\mathrm{SLL}/\hat{\mathrm{SLL}})$; E_P_, proxy exposure level; E_Pz_, proxy exposure *z*-score; ratio_z_, *z*-score for E_P_:SLL ratio; SLL, serum lipid level; $\hat{\mathrm{SLL}}$, predicted SLL. SLL predicted based on X_2_ in all scenarios. W not included in scenario D. In scenarios E and F, W = X_2_.	

*Results.* For scenario D (Figure 2), both standardization methods (2 and 6) and both covariate-adjusted standardization methods (3 and 7) performed well (Table 4). The remaining three methods were biased (absolute bias > 0.05) and showed sub-nominal CI coverage except when there was no true association (true OR = 1.0).

**Table 4:** Results from simulation studies comparing seven methods for serum lipid level adjustment when assessing the relationship between a serum biomarker and disease risk under different causal scenarios (Figure 2) and true effect sizes (true ORs = 2.0, 1.3, 1.0, 0.77, or 0.5).

Analysis method	Scenario D		Scenario E		Scenario F	
	Bias (SE)^*a*^	CI coverage	Bias (SE)^*a*^	CI coverage	Bias (SE)^*a*^	CI coverage
True OR = 2.0, true β for E_Tz_ = 0.650 (D and E) or 0.838 (F)
1. Unadjusted	–0.15 (0.003)	0.63	–0.04 (0.004)	0.92	0.03 (0.006)	0.94
2. Standardized^*b*^	0.01 (0.003)	0.95*	0.01 (0.003)	0.94*	0.01 (0.005)	0.94*
3. Covariate-adjusted standardization (CAS)^*b*^^,^^*c*^	0.01 (0.003)	0.94*	0.11 (0.004)	0.86	0.29 (0.006)	0.72
4. Covariate adjustment (CA)^*b*^	0.17 (0.004)	0.78	0.19 (0.004)	0.73	0.33 (0.007)	0.68
5. 2-stage model^*b*^	–0.12 (0.003)	0.73	–0.13 (0.004)	0.77	–0.02 (0.006)	0.92
6. Standardization plus CA^*b*^	0.01 (0.003)	0.94*	0.01 (0.003)	0.94*	0.01 (0.005)	0.94*
7. CAS plus CA^*b*^^,^^*c*^	0.01 (0.003)	0.94*	0.11 (0.004)	0.86	0.29 (0.006)	0.72
True OR = 1.3, true β for E_Tz_ = 0.245 (D and E) or 0.316 (F)
1. Unadjusted	–0.05 (0.002)	0.90	–0.01 (0.002)	0.95*	0.01 (0.003)	0.96*
2. Standardized^*b*^	0.00 (0.002)	0.94*	0.00 (0.002)	0.94*	0.00 (0.003)	0.96*
3. CAS^*b*^^,^^*c*^	0.00 (0.002)	0.95*	0.04 (0.002)	0.93	0.09 (0.004)	0.89
4. CA^*b*^	0.06 (0.003)	0.91	0.06 (0.003)	0.88	0.10 (0.004)	0.88
5. 2-stage model^*b*^	–0.05 (0.002)	0.90	–0.05 (0.002)	0.91	–0.02 (0.003)	0.94*
6. Standardization plus CA^*b*^	0.00 (0.002)	0.95*	0.00 (0.002)	0.94*	0.00 (0.003)	0.96*
7. CAS plus CA^*b*^^,^^*c*^	0.00 (0.002)	0.95*	0.04 (0.002)	0.93	0.09 (0.004)	0.89
True OR = 1.0, true β for E_Tz_ = 0.0
1. Unadjusted	0.01 (0.002)	0.96*	0.01 (0.002)	0.96*	0.00 (0.003)	0.96*
2. Standardized^*b*^	0.00 (0.002)	0.95*	0.00 (0.002)	0.95*	0.00 (0.003)	0.96*
3. CAS^*b*^^,^^*c*^	0.00 (0.002)	0.95*	0.00 (0.002)	0.96*	0.00 (0.003)	0.97
4. CA^*b*^	0.00 (0.003)	0.95*	0.00 (0.003)	0.95*	0.00 (0.003)	0.97
5. 2-stage model^*b*^	0.01 (0.002)	0.96*	0.01 (0.002)	0.96	0.01 (0.003)	0.96*
6. Standardization plus CA^*b*^	0.00 (0.002)	0.95*	0.00 (0.002)	0.95*	0.00 (0.003)	0.96*
7. CAS plus CA^*b*^^,^^*c*^	0.00 (0.002)	0.95*	0.00 (0.002)	0.95*	0.00 (0.003)	0.97
True OR = 0.77, true β for E_Tz_ = –0.245 (D and E) or –0.316 (F)
1. Unadjusted	0.06 (0.002)	0.85	0.02 (0.002)	0.95*	0.00 (0.003)	0.96*
2. Standardized^*b*^	0.00 (0.002)	0.95*	0.00 (0.002)	0.95*	0.00 (0.003)	0.95*
3. CAS^*b*^^,^^*c*^	0.00 (0.002)	0.95*	–0.03 (0.002)	0.93	–0.07 (0.003)	0.91
4. CA^*b*^	–0.06 (0.003)	0.90	–0.06 (0.003)	0.89	–0.08 (0.003)	0.90
5. 2-stage model^*b*^	0.06 (0.002)	0.86	0.07 (0.002)	0.87	0.05 (0.003)	0.92
6. Standardization plus CA^*b*^	0.00 (0.002)	0.95*	0.00 (0.002)	0.95*	0.00 (0.003)	0.95*
7. CAS plus CA^*b*^^,^^*c*^	0.00 (0.002)	0.95*	–0.03 (0.002)	0.93	–0.08 (0.003)	0.91
True OR = 0.5, true β for E_Tz_ = –0.650 (D and E) or –0.838 (F)
1. Unadjusted	0.17 (0.003)	0.55	0.06 (0.004)	0.90	0.01 (0.005)	0.94*
2. Standardized^*b*^	0.00 (0.003)	0.94*	–0.01 (0.003)	0.93	0.00 (0.004)	0.95*
3. CAS^*b*^^,^^*c*^	0.00 (0.003)	0.95*	–0.09 (0.004)	0.89	–0.22 (0.006)	0.78
4. CA^*b*^	–0.16 (0.004)	0.79	–0.17 (0.004)	0.77	–0.26 (0.006)	0.74
5. 2-stage model^*b*^	0.14 (0.003)	0.69	0.15 (0.004)	0.72	0.09 (0.005)	0.89
6. Standardization plus CA^*b*^	0.00 (0.003)	0.94*	–0.01 (0.003)	0.93	–0.01 (0.004)	0.95*
7. CAS plus CA^*b*^^,^^*c*^	0.00 (0.003)	0.94*	–0.09 (0.004)	0.89	–0.23 (0.006)	0.77
Abbreviations: E_Tz_, target-tissue exposure *z*-score; E_Pz_, proxy exposure *z*-score; SE, standard error; CI, confidence interval. Each simulation was repeated 1,000 times. Samples included 500 observations when the true OR = 2.0 or 0.5 and 1,000 observations otherwise. ^***a***^Bias is equal to the mean observed beta coefficient for β_Pz_, which is either the serum exposure *z*-score (Methods 1, 4, 5) or the *z*-score for the serum exposure to lipid level ratio (Methods 2, 3, 6), minus the true beta coefficient for E_Tz_. The standard deviation of the bias estimate is the square root of the average variance of β_Pz_ divided by the square root of the number of simulations. ^***b***^E and F are adjusted for X. ^***c***^Serum lipid levels are predicted using X. *CI coverage is consistent with 0.95 (0.95 ± 0.0135). Note that CI coverage values are rounded, and only those values that are consistent with 95% have been marked with an asterisk.						

When X influenced SLL and disease risk, as in scenario E (Figure 2), standardization (Method 2) and standardization plus covariate adjustment (Method 6) demonstrated little to no bias (± 0.01). The other methods were biased, particularly the covariate adjustment model (Method 4, bias = 0.19 when true OR = 2.0 and –0.17 when true OR = 0.5). The two covariate-adjusted standardization methods (3 and 7) were moderately biased when the effect was small (true OR = 1.3 or 0.77), and these methods performed more poorly when the effect size was large. For scenario F (Figure 2), the standardization methods (2 and 6) again showed the least bias and best CI coverage when X also affected E_O_.

In contrast to the results for the urinary biomarker scenarios, Methods 1 and 5 were biased when there was no true effect. The CI coverages were consistent with 95% for all methods and all scenarios. When classical assay measurement error was present [see Supplemental Material, Table S4, “Results from simulations with measurement error: serum biomarker scenarios (D–F)],” all methods suffered, but Methods 2 and 6 continued to have the best overall performance.

In general, when assessing the relationship between a health outcome and a lipid-soluble chemical measured in serum, we found that Methods 2 and 6 performed best. These methods involved standardizing the biomarker measurement by dividing it by the measured SLL. Method 6, in which the SLL was also included as a covariate, may be best suited for use in epidemiologic studies involving many interrelated covariates.

## Part III: Applied Example of Phthalates And Early Pregnancy Loss

*Methods.* We examined the association between mono-(3-carboxypropyl) phthalate (MCPP) and early pregnancy loss using data from the North Carolina Early Pregnancy Study (1982–1986). Details of the study have been described (Jukic et al. 2015; Wilcox et al. 1988). MCPP, human chorionic gonadotropin (hCG), and creatinine were measured in first-morning urine samples from 221 healthy women who were trying to conceive. MCPP and creatinine were assessed in specimens composed of three pooled, equal-volume aliquots collected during participants’ conception cycles (*n* = 198).

Conception was inferred if hCG concentrations were > 0.025 ng/mL on 3 consecutive days. A decline in hCG before 6 completed weeks (starting at the time of a woman’s last menstrual period) was considered an early pregnancy loss (*n* = 48). We considered the following variables as potential confounders on the basis of their possible relationship with early pregnancy loss and MCPP: age at conception, BMI, current smoking status, alcohol intake, caffeine intake, and education. Of these, only age was associated with creatinine in our study sample. Therefore, the DAG for this example would most resemble the previously described DAG C, with factor X = age and BMI, smoking, alcohol, caffeine, and education acting as confounders that are associated with MCPP (E_O_) and early loss but not predictive of creatinine.

We assessed the relationship between MCPP and early pregnancy loss using the seven statistical methods considered above. Because exposure units differ across methods, we used *z*-scores to allow comparison, although this scaling would not be used in applied settings where the investigator requires a unit-based effect measure.

*Results.* The median creatinine and MCPP concentrations were 1.4 g/L and 13.5 μg/L, respectively, with interquartile ranges of 1.1–1.7 and 9.5–21.1, respectively, and SDs of 0.5 and 13.5, respectively. Unadjusted, log-transformed MCPP and log-transformed creatinine were positively correlated (Pearson’s *r*^2^ = 0.28, *p* < 0.001). After creatinine standardization, logMCPP had a median of 2.32 μg/g creatinine (or 10.2 μg/g when exponentiated) and reduced variability [coefficient of variation (CV) = 0.26 vs. 0.27 when unstandardized, with CVs based on log-transformed values]. Variability was further reduced when we applied covariate-adjusted standardization before log-transformation (CV = 0.23).

Although none of the resulting ORs and 95% CIs indicate a statistically significant association between MCPP and early pregnancy loss, the estimates for Methods 1, 4, and 5 suggest a positive association, whereas the estimates for Methods 2, 3, 6, and 7 are < 1 (Table 5). If these same point estimates were reported for a study with a larger sample size, the choice of creatinine-adjustment approach could influence the conclusion.

**Table 5:** Odds ratios and 95% confidence intervals for the effect of mono-(3-carboxypropyl) phthalate (MCPP, as a *z*-score) on early pregnancy loss.

Analysis method	Odds ratio (95% confidence interval)
1. Unadjusted	1.16 (0.82, 1.62)
2. Standardized	0.95 (0.65, 1.39)
3. Covariate-adjusted standardization (CAS)	0.95 (0.65, 1.39)
4. Covariate adjustment (CA)	1.07 (0.72, 1.59)
5. 2-stage model	1.16 (0.82, 1.63)
6. Standardization plus CA	0.95 (0.65, 1.40)
7. CAS plus CA	0.95 (0.65, 1.40)
All models were adjusted for age, BMI, current smoking, alcohol intake, caffeine intake, and education. Creatinine was predicted using age only.	

In this example, we used pooled urine specimens to decrease the influence of short-term variations in hydration and to provide a stable assessment of phthalate concentrations over time. Although this was an important strength of the design, and pooling of multiple samples should be considered when feasible, it made the relative benefits of our novel standardization methods less apparent. Another limitation of this example is that the women included in the Early Pregnancy Study were fairly homogenous for factors associated with creatinine, including race (95% white), age (range 21–42), and BMI (89% were < 25 kg/m^2^).

## Discussion

When urinary concentrations of an environmental contaminant are used as surrogates for concentrations in risk-relevant target tissues, day-to-day and person-to-person variations in urine dilution can cause bias-inducing, power-eroding measurement error. Measurement error is also problematic in studies of lipophilic chemicals in serum, particularly if fasting serum samples are unavailable and it becomes impossible to tease out the relative influences of general adiposity versus those of recent fat intake on the measurements. Additional complexities arise for both biologic matrices if there are factors that influence both creatinine excretion and the outcome, or both SLL and the outcome.

Although the methods are controversial, convenient approaches to reducing measurement error bias for exposure biomarkers measured in urine and serum involve dividing by specific gravity or creatinine levels for urinary concentrations, or by a serum lipid summary measure for serum concentrations (Method 2) (Barr et al. 2005; Schisterman et al. 2005; Thorne 2008). With these standardization methods, we implicitly assume a causal model such as those shown in scenarios A and D, and the results from our simulations support the belief that standardization by division works well under these scenarios.

Concerns that traditional standardization is not appropriate in settings where disease risk factors can also affect creatinine (scenarios B and C) led us to devise a modified method. First, we model creatinine in relation to other known risk factors and obtain a predicted value for creatinine. The remaining proportional variation around the fitted mean is approximately attributable to hydration levels, which also affect the concentration of the biomarker of interest. We adjust the concentration in the urine by dividing by the ratio of the measured value to the fitted mean value of creatinine (Method 3). We also considered augmenting Method 3 by including adjustment for creatinine in the regression model for risk (Method 7). These analytic approaches performed well under scenarios B and C, in which a confounder of the exposure–disease association also influenced creatinine levels. On the basis of these results, we recommend that Method 7 be used for studies of urinary biomarkers that resemble the scenarios described in DAGs A, B, or C, and we provide code to implement this method in SAS (see Supplemental Material, “Part III: SAS coding example for implementation of covariate-adjusted standardization method”; http://www.niehs.nih.gov/research/resources/software/biostatistics/covariate/index.cfm).

The usefulness of this proposed method depends on the availability of relevant predictors because more informative prediction models for creatinine will improve measures of the residual proportion of creatinine attributable to hydration. Many predictors of creatinine, including age, race, sex, and BMI, are routinely collected, but the field could benefit from an improved understanding of physiologic factors that influence creatinine.

The issues associated with serum biomarker measures are more complex than those associated with urinary biomarker measures. Although creatinine and urinary E_P_ share a common causal ancestor (i.e., hydration), total SLL influences a lipophilic E_P_ directly. Additionally, total SLL is causally downstream from long-term adiposity, which is a risk factor for many chronic diseases. By contrast, a causal link between hydration/creatinine and disease (except kidney disease) seems unlikely, given that creatinine is a byproduct of muscle catabolism. Because of these discrepancies, we cannot use covariate-adjusted standardization to isolate the effects of SLL in the same way that we can approximately isolate the effects of hydration in the urinary biomarker examples.

In our simulations for the serum biomarker setting, the effect estimates were typically more biased than those for the urinary biomarker measurement scenarios. We found that the traditional standardization approach (Method 2) outperformed the covariate-adjusted standardization approach (Method 3), but we believe that in scenarios that are more complex than the ones simulated here, additionally including SLL as a covariate in the regression model (Method 6) will help to ensure that any backdoor paths are sufficiently blocked and that confounding is controlled. Because such adjustment may be useful even when SLL (or creatinine) is not acting as a confounder, it may be useful to think of SLL (or creatinine) as a “concomitant variable” or a non-confounding covariate that can improve estimation precision if included in the data analysis (Li et al. 2013).

A key feature of the causal diagrams presented herein is that we allow for the possibility that concentrations differ across tissues. For both the hypothetical and applied examples we have presented, we assume that urinary excretion concentrations are correlated with chemical concentrations in the target tissues but are not perfect surrogates for them. This conceptualization of the problem differs from that of Schisterman et al. (2005), which sometimes assumes that urinary or serum biomarker concentrations are directly causally related to the outcome. Moreover, we consider situations where SLL directly affects the amount of analyte present in the serum.

To enable meaningful comparisons of estimated beta coefficients across all seven models, we calculated *z*-scores and estimated the effects per SD increase in biomarker concentration. Unlike crude exposure measures, these *z*-scores are scale-invariant and thus allowed us to make direct comparisons of estimates derived using different methods. However, we do not recommend the use of *z*-scores in practice because SDs may vary considerably across studies or population subgroups.

We also considered situations in which a covariate can affect both serum lipid (or urinary creatinine) levels and the outcome (scenarios B and E) or in which a covariate can affect exposure, serum lipid/creatinine levels, and the outcome (scenarios C and F). We believe that the DAGs included here capture the key features of pertinent scenarios, but there may be other relevant situations that have not been addressed by our simulations. For example, we have not considered scenarios in which the target tissue is exposed directly (e.g., airborne contaminants and lung disease) or in which the exposure of interest is internally produced (e.g., hCG). We also acknowledge that our assumption that the relationships between hydration and creatinine and between hydration and E_P_ are multiplicative is an approximation, however plausible.

Typically, DAGs are used to select the minimal set of adjustment covariates needed to control confounding and permit valid causal inference. Instead, we have used DAGs to guide our understanding of measure surrogacy and sources of measurement error in settings where urine and blood enable convenient proxy measurement of environmental agents or biomarkers of exposure. Our treatment of measurement error using DAGs is incomplete in the sense that the scenarios considered involve only a “snapshot” measure of an exposure that may have long-lasting effects. However, the most realistic goal of any standardization approach is to control bias due to short-term and risk-irrelevant influences on the measurement.

We note that our results apply specifically to etiologic studies that measure the association of environmental exposures with a health outcome. Other scientific applications, such as studies of hormone secretion patterns across menstrual cycles (Baird et al. 1997), might rely on within-person changes over time. For such studies, factors that influence long-term concentrations may not be relevant.

Nevertheless, we believe that the general framework we have developed has broad applicability. For example, one could consider dietary biomarkers (e.g., urinary sodium) or analyte concentrations measured in other body fluids, such as saliva, semen, or breast milk. Each case would require careful consideration of the relationships among the proxy tissue, the target tissue, and any factors that could influence relative concentrations or confound the exposure–disease relationship.

## Conclusion

We have proposed a new covariate-adjusted standardization method to adjust for creatinine when estimating the association between a health outcome and environmental chemicals or biomarkers measured in urine. For studies of lipophilic contaminants measured in serum, our results suggest that a different, more traditional standardization approach is appropriate. In both cases, also adjusting for creatinine or SLL as a covariate seems to provide additional benefits. Other recently proposed approaches, such as including creatinine or serum lipids as adjustment variables in statistical models or accounting for residuals from a stage-one predictive model, did not work well in our causal scenarios that regarded urine or blood as surrogates for target tissues. With the use of the proposed methods, our simulations illustrated that it is possible to control for variations in creatinine or SLL due to risk-irrelevant temporal perturbations. Improved methods for standardizing biomarker measures should enable improved estimation of the effects of environmental exposures on human health.

## Supplemental Material

(152 KB) PDF
