# Supplementary material for: Environmental Chemicals in Urine and Blood: Improving Methods for Creatinine and Lipid Adjustment
Source: Environ Health Perspect. 2015 Jul 24;124(2):220–7. doi: 10.1289/ehp.1509693 (PMC4749084; doi:10.1289/ehp.1509693)
Supplement: (152 KB) PDF [file ehp.1509693.s001.acco.pdf]

Environ Health Perspect
DOI: 10.1289/ehp.1509693

Note to Readers: *EHP* strives to ensure that all journal content is accessible to all readers. However, some figures and Supplemental Material published in *EHP* articles may not conform to 508 standards due to the complexity of the information being presented. If you need assistance accessing journal content, please contact ehp508@niehs.nih.gov. Our staff will work with you to assess and meet your accessibility needs within 3 working days.

Supplemental Material

Environmental Chemicals in Urine and Blood: Improving Methods for Creatinine and Lipid Adjustment

Katie M. O'Brien, Kristen Upson, Nancy R. Cook, and Clarice R. Weinberg

Table of Contents

Supplemental Material, Part I: Description of simulation study parameters for urine biomarker scenarios (DAGs A-C)

Table S1: Variable relationships, urinary biomarker scenarios (A-C)

Table S2: Results from simulations with measurement error: urinary biomarker scenarios (A-C)

Supplemental Material, Part II: Description of simulation study parameters for serum biomarker scenarios (DAGs D-F)

Table S3: Variable relationships, serum biomarker scenarios (D-F)

Table S4: Results from simulations with measurement error: serum biomarker scenarios (D-F)

Supplemental Material, Part III: SAS coding example for implementation of covariate-adjusted standardization method

References

Supplemental Material, Part I: Description of simulation study parameters for urine biomarker scenarios (DAGs A-C)

For the creatinine-related scenarios (Figure 1A-1C) we assumed a multiplicative relationship between hydration and urinary creatinine and between hydration and the proxy biomarker concentration (E_P). Thus, for example, if hydration is doubled, the creatinine concentration will be halved, as will the biomarker concentration. Because we wanted to select realistic distribution parameters, we generated values for the overall exposure (E_O), the target tissue concentration (E_T), and E_P so that E_P concentrations would resemble urinary BPA concentrations measured in female participants from the 2007-2008 and 2009-2010 NHANES (unadjusted geometric means = 1.97 μg/L and 1.73 μg/L, respectively) (Fourth National Report on Human Exposure to Environmental Chemicals, 2014) We generated exposures that were log-normally distributed by randomly drawing values for ln(E_O) from a normal distribution and then exponentiating them. For DAGs A and B, ln(E_O) were drawn from a normal distribution with a mean of 1.3 and a standard deviation (std) of 0.3. For DAG C, E_O was dependent on X_1, with ln(E_O) randomly drawn from a normal distribution with mean=0.8+0.01*X_1 and std=0.3. In the DAG, X_1 can be thought of as age, a factor that influences both the exposure and creatinine.

In all scenarios, the exposure concentrations in the target tissue (E_T) were lower than overall exposure concentrations (E_O), but perfectly correlated: E_T=0.8*E_O. For DAGs A-C, hydration levels were generated by exponentiating values randomly drawn from a normal distribution (mean= -0.3, std= 0.08, and discarding if not between 0 and 1). This distribution was chosen so that hydration was measured as a fraction (mean= 0.74, maximum= 1.0). X_1, which we can think of as age, was drawn from a normal distribution with mean=50 and std=10, discarding values below 18 and above 85.

In all scenarios, the proxy exposure concentration (E_P) was set to (0.37*E_O)/Hydration, which produced mean E_P of approximately 1.9 (consistent with NHANES). Under scenario A, creatinine concentration was simply a function of hydration (creatinine= 0.71/hydration). In scenarios B and C, creatinine was dependent on both hydration and X_1 (creatinine= (1.21-0.01*X_1)/hydration). Note that at the mean age (50 years), the creatinine distribution is the same as it was in the scenario A. In all three scenarios, creatinine-corrected E_P values (ratio= E_P/creatinine) had a distribution similar to that observed for BPA in NHANES.

We also considered scenarios in which E_P and creatinine were subject to random variation due to assay errors. We simulated this by adding random error terms to the equations used to generate E_P and creatinine. For E_P, the error term was normally distributed with mean= 0 and std= 0.2, and for creatinine the error terms were normally distributed with mean= 0 and std= 0.05.

Presence or absence of disease was determined by random draws from a binomial distribution, where the ln odds of having disease was dependent on the product of E_T and the ln odds ratio (OR) of the true effect (β_{TRUE}). We ran 5 sets of simulations, specifying true (target-tissue based) ORs of 2.00, 1.30, 1.00, 0.77 and 0.50. These correspond to β_{TRUE} values of 0.69, 0.26, 0, -0.26 and -0.69, respectively.

In scenarios B and C, X_1 also influenced disease risk, with ln OR= 0.05 per unit. If X_1 is age, this corresponds to an OR of 1.05 for each year increase. We selected intercept terms to impose case-control sampling and ensure that approximately 50% of the individuals would be cases.

Table S1: Variable relationships, urinary biomarker scenarios (A-C)

Parameter	Scenario A	Scenario B	Scenario C
**E_O**	$\ln(E_O) \sim N(1.3, 0.3^2)$ $\sigma_{E_O}{}^2 = \left(e^{\sigma^2_{lnE_O}} - 1\right)\left(e^{2\mu_{lnE_O}+\sigma^2_{lnE_O}}\right)$ $= (e^{0.3^2} - 1)(e^{2*1.3+0.3^2}) = 1.39$ $\sigma_{E_O} = 1.18$	$\ln(E_O) \sim N(1.3, 0.3^2)$ $\sigma_{E_O} = 1.18$	$\ln(E_{O*}) \sim N(0.8 + 0.01A, 0.3^2)$ $A \sim N(50,10)^{\dagger}$ $\sigma_{lnE_{O*}} = \sqrt{0.3^2 + 0.01^2 9.95^2} = 0.32$ $\sigma_{E_{O*}} = 1.25$
**E_T**	$0.8*E_O$	$0.8*E_O$	$0.8*E_{O*}$
**$E_{Tz} = \frac{E_T - \overline{E_T}}{\sigma_{E_T}}$**	$\frac{0.8E_O - 0.8\overline{E_O}}{\sqrt{0.8^2\sigma_{E_O}{}^2}} = \frac{E_O - \overline{E_O}}{\sigma_{E_O}}$	$\frac{E_O - \overline{E_O}}{\sigma_{E_O}}$	$\frac{E_{O*} - \overline{E_{O*}}}{\sigma_{E_{O*}}}$
**E_P**	$(0.37*E_O)/H = (0.37*E_O*C)/0.71 =$ $0.52*E_O*C$	$(0.37*E_O)/H =$ $(0.37*E_O*C)/(1.21-0.01*X_1)$ @X_1=50: $E_P = (0.52*E_O*C)$	$(0.37*E_{O*})/H =$ $(0.37*E_{O*}*C)/(1.21-0.01* X_1)$ @X_1=50: $E_P = (0.52*E_{O*}*C)$
**$E_{Pz} = \frac{E_P - \overline{E_P}}{\sigma_{E_P}}$**	$\frac{0.52E_OC - 0.52\overline{E_O}\bar{C}}{\sqrt{0.52^2\sigma_{E_O}{}^2\sigma_C{}^2}} = \frac{E_OC - \overline{E_O}\bar{C}}{\sigma_{E_O}\sigma_C}$	$\frac{E_OC - \overline{E_O}\bar{C}}{\sigma_{E_O}\sigma_C}$	$\frac{E_{O*}C - \overline{E_{O*}}\bar{C}}{\sigma_{E_{O*}}\sigma_C}$
**C**	$0.71/H$	$(1.21-0.01*X_1)/H$	$(1.21-0.01*X_1)/H$
**ratio (E_P/C)**	$0.52*E_O*C$ / $C = 0.52*E_O$	$(0.52*E_O*C)/C = 0.52*E_O$	$(0.52*E_{O*}*C)/C = 0.52*E_{O*}$
**$\text{ratio}_z = \frac{ratio - \overline{ratio}}{\sigma_{ratio}}$**	$\frac{0.52E_O - 0.52\overline{E_O}}{\sqrt{0.52^2\sigma_{E_O}{}^2}} = \frac{E_O - \overline{E_O}}{\sigma_{E_O}}$	$\frac{E_O - \overline{E_O}}{\sigma_{E_O}}$	$\frac{E_{O*} - \overline{E_{O*}}}{\sigma_{E_{O*}}}$
**D**	logit(Pr[D])= $\beta_0 + \beta_1 E_T$ $= \beta_0 + \beta_1(0.8 * E_O)$ $= \beta_0 + \beta_1(0.8 * (E_{Tz}\sigma_{E_O} + \overline{E_O})) =$ $\beta_0 + \beta_1(0.8 * (E_{Pz}\sigma_{E_O}\sigma_C + \overline{E_O C})/C)$ $= \beta_0 + \beta_1(0.8 * (\text{ratio}_z\sigma_{E_O} + \overline{E_O}))$	logit(Pr[D])= $\beta_0 + \beta_1(0.8 * E_O) +$ $\beta_2 X_1$ $= \beta_0 + \beta_1(0.8 * (E_{Tz}\sigma_{E_O} + \overline{E_O}))$ $+ \beta_2 X_1$ $= \beta_0 + \beta_1(0.8 * (E_{Pz}\sigma_{E_O}\sigma_C +$ $\overline{E_O C})/C) + \beta_2 X_1$ $= \beta_0 + \beta_1(0.8 * (\text{ratio}_z\sigma_{E_O} + \overline{E_O}))$ $+ \beta_2 X_1$	logit(Pr[D])= $\beta_0 + \beta_1(0.8 * E_{O*}) +$ $\beta_2 X_1$ $= \beta_0 + \beta_1(0.8 * (E_{Tz}\sigma_{E_{O*}} + \overline{E_{O*}}))$ $+ \beta_2 X_1$ $= \beta_0 + \beta_1(0.8 * (E_{Pz}\sigma_{E_{O*}}\sigma_C +$ $\overline{E_{O*} C})/C) + \beta_2 X_1$ $= \beta_0 + \beta_1(0.8 * (\text{ratio}_z\sigma_{E_O} + \overline{E_{O*}}))$ $+ \beta_2 X_1$
**β coefficients**	$E(\beta_{ETz}) = 0.8\beta_1\sigma_{Eo} = E(\beta_{ratioz})$	$E(\beta_{ETz}) = 0.8\beta_1\sigma_{Eo} = E(\beta_{ratioz})$[with adjustment for X_1]	$E(\beta_{ETz}) = 0.8\beta_1\sigma_{Eo*} = E(\beta_{ratioz})$ [with adjustment for X_1]

Abbreviations: E_O=Overall exposure (alternate definition for DAG C denoted E_{O*}), E_T= Target tissue exposure, E_{Tz}= Target tissue exposure z-score, E_P= Proxy biomarker level, E_{Pz}= Proxy biomarker z-score, H= Hydration, C= Creatinine, ratio_z= z-score for E_P/C, D= Disease

$^{\dagger}X_1$ truncated at 18 and 85; Mean=50.01; Standard Deviation=9.95

Table S2: Results from simulations with measurement error: urinary biomarker scenarios (A-C)

	A		B		C	
**Analysis method**	Bias[a]	CIC	Bias[a]	CIC	Bias[a]	CIC
**True OR= 1.3, True β for E_{Tz}=0.245 (A, B), E_{Tz} = 0.260 (C)**						
1.Unadjusted	-0.02	0.93	-0.02	0.94	-0.02	0.94
2. Standardized[b]	-0.02	0.93	0.00	0.95*	0.03	0.93
3. Covariate-adjusted standardization (CAS)[b,c]	-0.02	0.93	-0.02	0.94	-0.02	0.95*
4. Covariate adjustment (CA)[b]	-0.01	0.94*	-0.01	0.94*	-0.01	0.95*
5. 2-stage model[b]	-0.02	0.93	-0.02	0.94*	0.00	0.95*
6. Standardization plus CA[b]	-0.02	0.93	0.01	0.95*	0.03	0.93
7. CAS plus CA[b,c]	-0.02	0.93	-0.01	0.94*	-0.01	0.95*
**True OR= 0.77, True β for E_{Tz}= -0.245 (A, B), E_{Tz}= -0.260 (C)**						
1.Unadjusted	0.02	0.94*	0.02	0.94	0.03	0.93
2. Standardized[b]	0.02	0.94*	0.00	0.95*	-0.02	0.96*
3. CAS[b,c]	0.02	0.94*	0.02	0.94*	0.02	0.94*
4. CA[b]	0.01	0.95*	0.01	0.95*	0.02	0.94*
5. 2-stage model[b]	0.02	0.95*	0.02	0.94*	0.00	0.95*
6. Standardization plus CA[b]	0.01	0.95*	0.00	0.95*	-0.02	0.96*
7. CAS plus CA[b,c]	0.01	0.95*	0.01	0.94*	0.02	0.94*

Abbreviations: E_{Tz}= Target tissue exposure z-score, E_{Pz}= Proxy exposure z-score, CIC=Confidence Interval Coverage

Each simulation was repeated 1,000 times. Samples included 1,000 observations.

[a]Bias is equal to the mean observed beta coefficient for β_{Pz}, which is the beta for E_{Pz} (Methods 1, 4, 5), $ratio_z$ (Methods 2 and 6) or $Cratio_z$ (methods 3 and 7), minus the true beta coefficient for E_{Tz}. The standard deviation of the bias estimate is the square root of the average variance of β_{Pz} divided by the square root of the number of simulations.

[b]Scenarios B and C, are adjusted for X_1

[c]Creatinine levels predicted using X_1 (A,B,C)

*CIC is consistent with 0.95 (0.95±0.0135)

Supplemental Material, Part II: Description of simulation study parameters for serum biomarker scenarios (DAGs D-F)

Scenarios D, E, and F (Figures 2D, 2E, and 2F, respectively) were constructed in much the same way as A, B, and C, respectively. To simplify things and allow easier comparisons between the urine and blood-based examples, we used the same distributions to define E_O, E_P and E_T, even though these values were now conceptualized as representing PCB exposure, rather than BPA exposure. E_T concentrations were again set to 0.8*E_O for all DAGs. For DAGs D and E, E_O concentrations were generated by exponentiating values randomly drawn from a normal distribution (with mean= 1.3, std= 0.3).

In DAGs D-F, total serum lipid level (SLL) is the sum of two components: adiposity-related SLL and variable SLL. Adiposity-SLL is positively associated with body size, but stable within individuals. In contrast, variable-SLL is easily altered by recent fat intake (RFI). To create values for RFI, we randomly drew from a normal distribution with mean= 0.5 and std= 0.2, discarding values below 0 or above 1. For DAG D, we generated values for "variable-SLL" based on RFI alone (variable-SLL= -0.15+0.8*RFI+ε_1), where ε_1 was normally distributed with mean 0 and std 0.05. "Adiposity-related SLL" was generated by exponentiating random draws from a normal distribution (mean= -0.29, std= 0.25). In DAGs E and F, adiposity-related SLL was dependent on X_2, which can be thought of as BMI. X_2 was drawn from a random normal distribution with mean= 29 and std= 4.5, truncated to be greater than 16.5 kg/m^2. Therefore, at the mean BMI (29 kg/m^2), the adiposity-SLL distribution is the same as it was in the scenario D. Adiposity-related SLL was equal to -0.41+0.04*X_2+ε_2, where ε_2 was normally distributed with mean= 0 and std= 0.1. E_P was assumed to be a lipophilic chemical and have a multiplicative relationship with SLL and E_O through the relationship E_P=0.5*E_O*SLL. Accordingly, if SLL doubled, E_P would also double. For DAG F, E_O also depended on X_2. For a given value of X_2, ln E_O was drawn from a normal distribution with mean= -0.154+0.05*X_2 and std= 0.3.

We also considered scenarios in which E_P and SLL measurements were subject to random variation due to assay errors. We simulated this by adding random error terms to the equations used to generate E_P and SLL. For E_P, the error term was normally distributed with mean= 0 and std= 0.2, and for SLL the error terms were normally distributed with mean= 0 and std= 0.05.

Presence or absence of disease was determined by random draws from a binomial distribution, where the ln odds of having disease was dependent on the product of E_T and the ln odds ratio (OR) of the true effect (β_{TRUE}). We ran 5 sets of simulations, specifying true (target-tissue based) ORs of 2.00, 1.30, 1.00, 0.77 and 0.50. These correspond to β_{TRUE} values of 0.69, 0.26, 0, -0.26 and -0.69, respectively.

In scenarios E and F, X_2 affected disease risk with ln OR= 0.10 per unit. If X_2 is BMI, this corresponds to an OR of 1.11 per kg/m^2 increase in BMI. SLL were also associated with disease (ln OR= 0.10 per unit). We selected intercept terms to impose case-control sampling and ensure that approximately 50% of the individuals would be cases.

Table S3: Variable relationships, serum biomarker scenarios (D-F)

Parameter	Scenario D	Scenario E	Scenario F
**E_O**	$\ln(E_O) \sim N(1.3, 0.3^2)$<br>${\sigma_{E_O}}^2 = \left(e^{\sigma^2_{lnE_O}} - 1\right)\left(e^{2\mu_{lnE_O}+\sigma^2_{lnE_O}}\right)$<br>$= \left(e^{0.3^2} - 1\right)\left(e^{2*1.3+0.3^2}\right) = 1.39$<br>$\sigma_{E_O} = 1.18$	$\ln(E_O) \sim N(1.3, 0.3^2)$<br>$\sigma_{E_O} = 1.18$	$\ln(E_{O*}) \sim N(-0.154 + 0.05B, 0.3^2)$<br>$B \sim N(29,4.5)^{\dagger}$<br>$\sigma_{lnE_{O*}} = \sqrt{0.3^2 + 0.05^2 4.45^2} = 0.37$<br>$\sigma_{E_{O*}} = 1.52$
**E_T**	$0.8*E_O$	$0.8*E_O$	$0.8*E_{O*}$
**$E_{Tz} = \frac{E_T - \overline{E_T}}{\sigma_{E_T}}$**	$\frac{0.8E_O - 0.8\overline{E_O}}{\sqrt{0.8^2 {\sigma_{E_O}}^2}} = \frac{E_O - \overline{E_O}}{\sigma_{E_O}}$	$\frac{E_O - \overline{E_O}}{\sigma_{E_O}}$	$\frac{E_{O*} - \overline{E_{O*}}}{\sigma_{E_{O*}}}$
**E_P**	$0.5*E_O*S$	$0.5*E_O*S$	$0.5*E_{O*}*S$
**$E_{Pz} = \frac{E_P - \overline{E_P}}{\sigma_{E_P}}$**	$\frac{0.5E_O S - 0.5\overline{E_O S}}{\sqrt{0.55^2 {\sigma_{E_O}}^2 {\sigma_S}^2}} = \frac{E_O S - \overline{E_O S}}{\sigma_{E_O}\sigma_S}$	$\frac{E_O S - \overline{E_O S}}{\sigma_{E_O}\sigma_S}$	$\frac{E_{O*} S - \overline{E_{O*} S}}{\sigma_{E_{O*}}\sigma_S}$
**S**	S_A-0.15+0.8*R	-0.56+0.04*X_2+0.8*R	-0.56+0.04*X_2+0.8*R
**ratio (E_P/S)**	$0.5*E_O$	$0.5*E_O$	$0.5*E_{O*}$
**$ratio_z = \frac{ratio - \overline{ratio}}{\sigma_{ratio}}$**	$\frac{0.5E_O - 0.5\overline{E_O}}{\sqrt{0.5^2 {\sigma_{E_O}}^2}} = \frac{E_O - \overline{E_O}}{\sigma_{E_O}}$	$\frac{E_O - \overline{E_O}}{\sigma_{E_O}}$	$\frac{E_{O*} - \overline{E_{O*}}}{\sigma_{E_{O*}}}$
**D**	logit(Pr[D])= $\beta_0 + \beta_1 E_T + \beta_2 S_A$<br>$= \beta_0 + \beta_1(0.8 * E_O) + \beta_2 S_A$<br>$= \beta_0 + \beta_1(0.8 * (E_{Tz}\sigma_{E_O} + \overline{E_O}))$<br>$+ \beta_2 S_A$<br>$=\beta_0 + \beta_1(0.8 * (E_{Pz}\sigma_{E_O}\sigma_S +$<br>$\overline{E_O}\bar{S})/S) + \beta_2 S_A$<br>$= \beta_0 + \beta_1(0.8 * ratio_z\sigma_{E_O} +$<br>$\overline{E_O}) + \beta_2 S_A$	logit(Pr[D])= $\beta_0 + \beta_1(0.8 * E_O) +$<br>$\beta_2 S_A + \beta_3 X_2$<br>$= \beta_0 + \beta_1(0.8 * (E_{Tz}\sigma_{E_O} + \overline{E_O}))$<br>$+ \beta_2 S_A + \beta_3 X_2$<br>$=\beta_0 + \beta_1(0.8 * (E_{Pz}\sigma_{E_O}\sigma_S +$<br>$\overline{E_O}\bar{S})/S) + \beta_2 S_A + \beta_3 X_2$<br>$= \beta_0 + \beta_1\left(0.8 * ratio_z\sigma_{E_O} + \overline{E_O}\right) +$<br>$\beta_2 S_A + \beta_3 X_2$	logit(Pr[D])= $\beta_0 + \beta_1(0.8 * E_{O*}) +$<br>$\beta_2 S_A + \beta_3 X_2$<br>$= \beta_0 + \beta_1(0.8 * (E_{Tz}\sigma_{E_{O*}} +$<br>$\overline{E_{O*}})) + \beta_2 S_A + \beta_3 X_2$<br>$=\beta_0 + \beta_1(0.8 * (E_{Pz}\sigma_{E_{O*}}\sigma_S +$<br>$\overline{E_O}\bar{S})/S) + \beta_2 S_A + \beta_3 X_2$<br>$= \beta_0 + \beta_1\left(0.8 * ratio_z\sigma_{E_{O*}} + \overline{E_{O*}}\right)$<br>$+ \beta_2 S_A + \beta_3 X_2$
**β coefficients**	$E(\beta_{ETz})= 0.8\beta_1\sigma_{Eo} = E(\beta_{ratioz})$ [assuming adequate adjustment for S_A]	$E(\beta_{ETz})= 0.8\beta_1\sigma_{Eo} = E(\beta_{ratioz})$ [assuming adequate adjustment for S_A and X_2]	$E(\beta_{ETz})= 0.8\beta_1\sigma_{Eo} = E(\beta_{ratioz})$ [assuming adequate adjustment for S_A and X_2]

Abbreviations: E_O=Overall exposure (alternate definition for DAG F denoted E_{O*}), E_T= Target tissue exposure, E_{Tz}= Target tissue exposure z-score, E_P= Proxy biomarker level, E_{Pz}= Proxy biomarker z-score, S= Serum lipid level, S_V= Variable serum lipid level, S_A= Adiposity serum lipid level R= Recent fat intake, $ratio_z$= z-score for E_P/S, D= Disease

$^{\dagger}X_2$ truncated to be >16.5; Mean=29.04; Standard Deviation=4.45

Table S4: Results from simulations with measurement error: serum biomarker scenarios (D-F)

	D		E		F	
**Analysis method**	Bias[a]	CIC	Bias[a]	CIC	Bias[a]	CIC
**True OR = 1.3, True β for E_{Tz}= 0.245 (D, E), E_{Tz} = 0.316 (F)**						
1.Unadjusted	-0.05	0.87	-0.02	0.94	-0.01	0.95*
2. Standardized[b]	-0.02	0.94*	-0.02	0.94*	-0.03	0.93
3. Covariate-adjusted standardization (CAS)[b,c]	-0.02	0.94*	0.01	0.94*	0.05	0.94*
4. Covariate adjustment (CA)[b]	0.03	0.95*	0.04	0.93	0.06	0.93
5. 2-stage model[b]	-0.05	0.88	-0.05	0.90	-0.04	0.93
6. Standardization plus CA[b]	-0.02	0.95*	-0.02	0.94*	-0.03	0.94
7. CAS plus CA[b,c]	-0.02	0.95*	0.01	0.94*	0.05	0.94*
**True OR = 0.77, True β for E_{Tz}= -0.245 (D, E), E_{Tz} = -0.316 (F)**						
1.Unadjusted	0.07	0.83	0.03	0.93	0.02	0.95*
2. Standardized[b]	0.02	0.94*	0.02	0.93	0.03	0.93
3. CAS[b,c]	0.02	0.94*	-0.01	0.95*	-0.04	0.94*
4. CA[b]	-0.03	0.94*	-0.03	0.93	-0.05	0.93
5. 2-stage model[b]	0.07	0.83	0.07	0.86	0.06	0.90
6. Standardization plus CA[b]	0.02	0.95*	0.02	0.93	0.03	0.93
7. CAS plus CA[b,c]	0.02	0.95*	-0.01	0.95*	-0.04	0.94*

Abbreviations: E_{Tz}= Target tissue exposure z-score, E_{Pz}= Proxy exposure z-score, CIC=Confidence Interval Coverage
Each simulation was repeated 1,000 times. Samples included 1,000 observations.

[a]Bias is equal to the mean observed beta coefficient for β_{Pz}, which is the beta for E_{Pz} (Methods 1, 4, 5), $ratio_z$ (Methods 2 and 6) or $Cratio_z$ (methods 3 and 7), minus the true beta coefficient for E_{Tz}. The standard deviation of the bias estimate is the square root of the average variance of β_{Pz} divided by the square root of the number of simulations.

[b]Scenarios E and F are adjusted for X_2

[c]Serum lipid levels predicted using X_2 (D,E,F)

*CIC is consistent with 0.95 (0.95±0.0135)

Supplemental Material, Part III: SAS coding example for implementation of covariate-adjusted standardization method

(also at: http://www.niehs.nih.gov/research/resources/software/biostatistics/covariate/index.cfm)

For urinary biomarker analyses where creatinine has been measured at the same time as the biomarker of interest, the covariate-adjusted standardization plus creatinine covariate-adjustment approach performed well. To implement this approach, we first modeled the relationship between log creatinine (logC) and factors known to influence creatinine (e.g. age) using linear regression. This estimates the quantity of creatinine attributable to known factors. The 'OUTPUT' statement is used to save the predicted log creatinine values (plogC) in the 'pred' file.

```
DATA data;
     SET data;
     logC=log(C);
RUN;

PROC REG data=data;
     MODEL logC=age;
     OUTPUT out=pred p=plogC;
RUN;
```

Because the predicted value accounts for known determinants of creatinine, division of the measured contaminant concentration (E) by the creatinine ratio (observed divided by predicted creatinine) should, theoretically, produce an error-corrected measure of the individual's exposure level attributable to hydration alone. This is useful because hydration also directly affects the contaminant of interest. Note that we have to exponentiate the predicted log creatinine value to get the predicted creatinine value before calculating the covariate-adjusted exposure value (here E_crt).

```
DATA data;
     MERGE data pred;
     BY ID; *files should be sorted by ID;
     pC=exp(plogC);
     Cratio=C/pC;
     E_crt=E/Cratio;
RUN;
```

We can still include creatinine (C) as a covariate in the exposure-outcome regression model to control residual confounding. The adjustment model should include the covariates that

affect creatinine in addition to any other confounders. These other confounders are denoted Z1 and Z2. The outcome is disease (D).

```
PROC LOGISTIC data=data DESC;
     MODEL D= E_crt age Z1 Z2 C;
RUN;
```

References

Fourth National Report on Human Exposure to Environmental Chemicals. http://www.cdc.gov/exposurereport/ Accessed January 14, 2014.
